# Removal of Deeply Impacted Mandibular Molars by Sagittal Split Osteotomy

**DOI:** 10.1155/2016/1902089

**Published:** 2016-06-27

**Authors:** Erol Cansiz, Sabri Cemil Isler, B. Alper Gultekin

**Affiliations:** ^1^Department of Oral and Maxillofacial Surgery, Istanbul University, Faculty of Dentistry, 34104 Istanbul, Turkey; ^2^Department of Oral Implantology, Istanbul University, Faculty of Dentistry, 34104 Istanbul, Turkey

## Abstract

Mandibular third molars are the most common impacted teeth. Mandibular first and second molars do not share the same frequency of occurrence. In rare cases the occlusal surfaces of impacted molars are united by the same follicular space and the roots pointing in opposite direction; these are called kissing molars. In some cases, a supernumerary fourth molar can be seen as unerupted and, in this case, such a supernumerary, deeply impacted fourth molar is seen neighboring kissing molars. The extraction of deeply impacted wisdom molars from the mandible may necessitate excessive bone removal and it causes complications such as damage to the inferior alveolar nerve and iatrogenic fractures of the mandible. This case report describes the use of the sagittal split osteotomy technique to avoid extensive bone removal and protect the inferior alveolar nerve during surgical extruction of multiple impacted teeth.

## 1. Introduction

The removal of deeply impacted molars presents a significant challenge especially when the multiple impaction is present. Due to the location of the impacted teeth the surgical approach can be modified [1]. In this case a supernumerary molar joining kissing molar complicates the surgery. Common techniques such as buccal corticotomy/osteotomy, lingual split, and extraoral access are used for the surgical extruction of the complicated multiple impaction [2]. Unfortunately, they involve the loss of adjacent teeth, jaw fracture, TMJ disorders, and damage to the lingual or inferior alveolar nerves [3, 4]. In addition, extraoral approach may also result in scar formation on skin and has potential risk of injuring the marginal mandibular branch of the facial nerve [5].

Unlike the conventional procedures, the use of SSO for the teeth extruction can reduce the risk of these potential complications. Although sagittal split osteotomy is an orthognathic surgery procedure, it allows excellent access to the impacted teeth in the middle of the ascending ramus [6] and simultaneously avoids excessive bone removal [6, 7]. This case describes the technique and points out the value of sagittal split osteotomy in the removal of multiple deeply impacted mandibular molars.

## 2. Case Report

**Figure 1:**
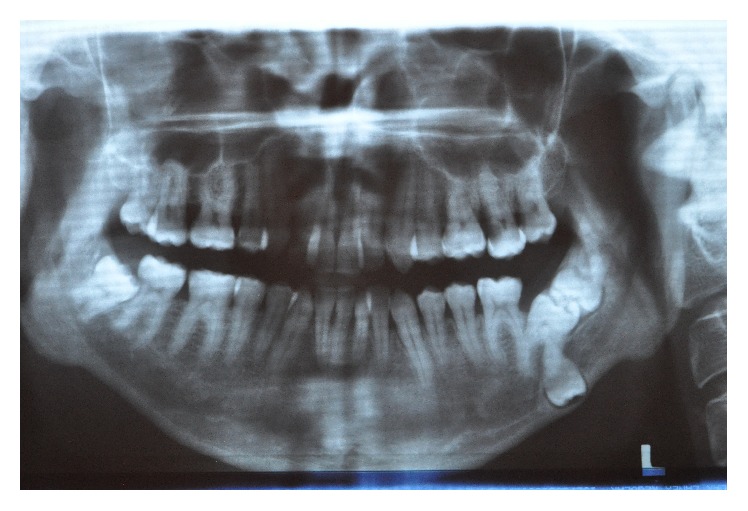
Preoperative panoramic radiography.

**Figure 2:**
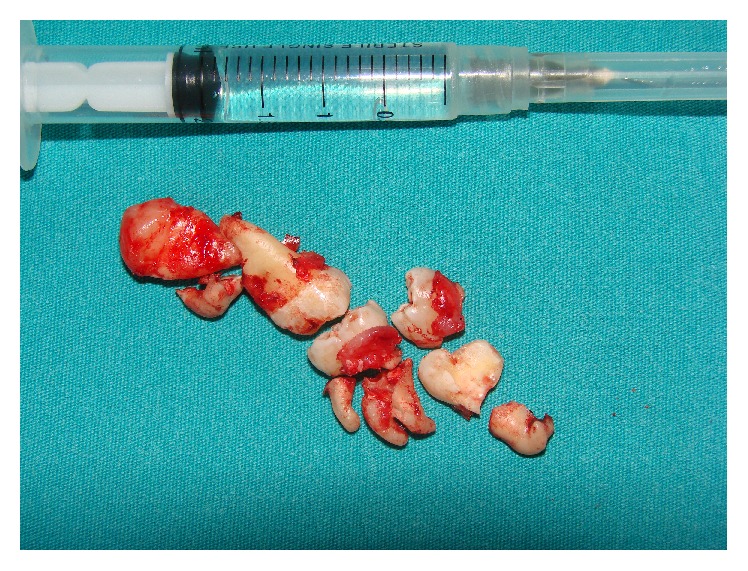
Split and removed impacted teeth.

**Figure 3:**
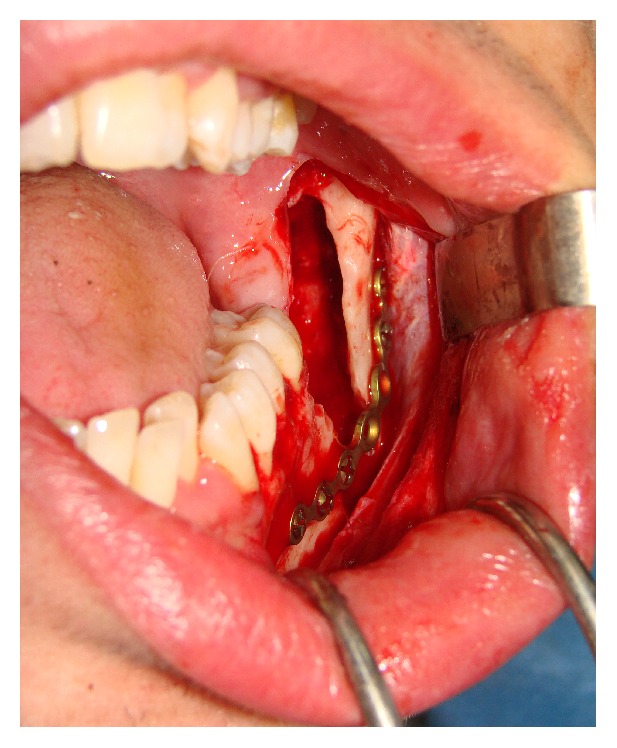
Postoperative intraoral view. Rigid fixation of split segments of the mandible.

**Figure 4:**
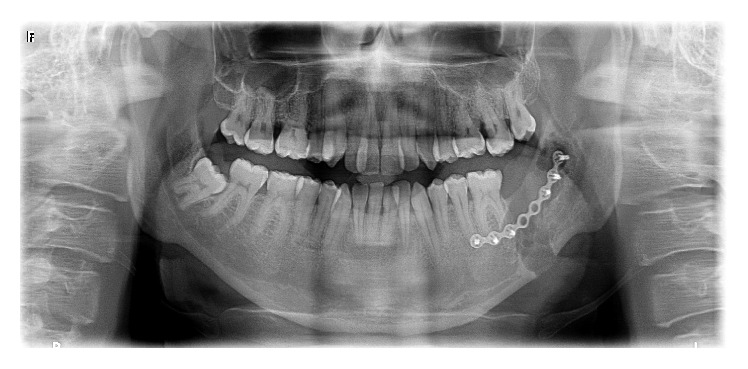
Postoperative panoramic radiography showing rigid fixation of the segments and complete removal of impacted teeth.

**Figure 5:**
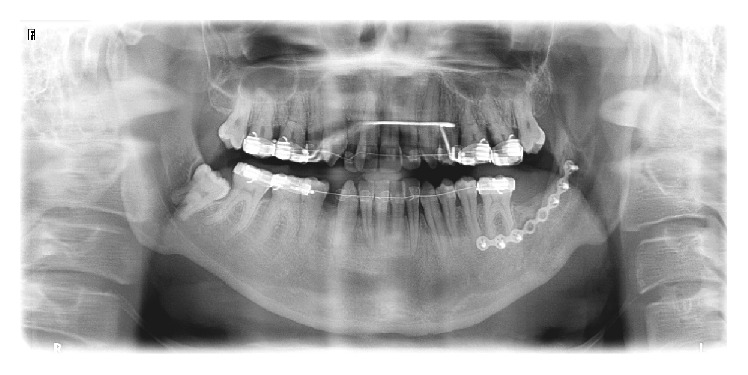
Postoperative control radiography after 6 months of healing period.

A 21-year-old male systemically healthy patient was referred to our department for the extraction of impacted molars identified during radiologic examination before orthodontic treatment (Figure 1). After the intraoral and radiologic examination it was decided that the impacted teeth are to be extracted by using sagittal split osteotomy. A conventional technique would require an extensive removal of alveolar bone and add the potential risk of damaging the inferior alveolar nerve; we decided that the tooth should be removed through a sagittal split of the mandible, which resulted in a favourable split of the mandibular ramus. Under intravenous sedation and local anesthesia, a diagonal incision starting from retromolar region and extending to canine tooth was performed and the full thickness flap was raised. A horizontal osteotomy was performed on the medial wall of the ramus 5 mm above the mandibular foramina. Then, the vertical osteotomy was done at the distal border of second molar. Finally, the vertical and the horizontal osteotomies were connected by oblique osteotomy on the level of external oblique line. The oblique osteotomy was performed with a 2 mm Lindeman burr to expose the impacted teeth and to facilitate the extraction. The teeth were separated and extracted piece by piece in order to protect the bone (Figure 2). After the extraction process, proximal and distal segments were fixed by using an eight-hole miniplate and 6 miniscrews (Figures 3 and 4). The flap was closed primarily and the healing period was entirely successful except that there was a temporary paresthesia of the inferior alveolar nerve which soon recovered after 6 months (Figure 5).

## 3. Discussion

In the absence of any well-defined operation procedures, the extraction of kissing molars combined with supernumerary teeth challenges the surgeon. The depth of the impacted teeth determines the degree of difficulty in their extraction. There are a variety of different surgical techniques to extract deeply impacted molars such as the buccal osteotomy, lingual split, and extraoral approach [2]. Rather than these conventional techniques that require extensive bone removal to gain access to impacted teeth, a unilateral SSO technique, primarily used in orthognathic surgery, was performed in this case.

The conventional removal of impacted teeth results in 1.6–3.3% inferior alveolar nerve paralysis and 0.9–11% lingual nerve paresthesia in all cases [8]. In addition, the extraoral approach jeopardizes the marginal mandibular branch of the facial nerve and may cause scar formation [3–5].

The sagittal split osteotomy (SSO) may prove to be a useful extraction method of deeply impacted mandibular molars due to its controlled manner of removing bone and its reduction of the risk of alveolar nerve damage via the direct identification of anatomic structures. On the other hand, temporary paresthesia incidence of the IAN is high but recovers within approximately 6–12 months [9].

As the buccal cortex is generally quite thin, the SSO becomes complicated in the presence of impacted teeth, which may lead to a bad split or an unfavorable mandibular fracture, and multiple impaction further worsens the SSO in this case [10]. To overcome this difficulty, the routine SSO procedure was modified by burring with a 2 cm Lindemann burr rather than chiseling and malleting technique [11].

In order to prevent an unfavorable fracture at the proximal segment, the vertical osteotomy must be properly extended to the mandibular base and any extensive bone removal surrounding impacted teeth must be avoided until the split of the buccal cortex is completed [6, 7].

When the IAN stays at the buccal cortex after the splitting procedure, it may be better to dissect the nerve to protect it as the compression that occurs during the elevation or sectioning of the teeth can cause damage. In our case, the IAN stayed at the lingual segment so the nerve was not dissected. However, temporary paresthesia occurred for 6 months regardless. In conclusion, the use of SSO is a useful technique that increases the safety of the removal of multiple deeply impacted teeth.

Although conventional osteotomy techniques were used in this case, it may be beneficial to use piezosurgery instrument to protect the IAN. In addition, using piezosurgery instrument may provide more controlled osteotomy and less bone loss than burring technique. However, using piezosurgery may increase total operation duration.
